# The regulatory role of abscisic acid on cadmium uptake, accumulation and translocation in plants

**DOI:** 10.3389/fpls.2022.953717

**Published:** 2022-09-13

**Authors:** Chuang Shen, Yu-Mo Yang, Ying-Fang Sun, Man Zhang, Xiao-Jing Chen, Ying-Ying Huang

**Affiliations:** Research Center for Environmental Pollution Control Technology, School of Chemical and Environmental Engineering, Hunan Institute of Technology, Hengyang, China

**Keywords:** ABA synthesis, Cd uptake, Cd translocation, root development, ROS

## Abstract

To date, Cd contamination of cropland and crops is receiving more and more attention around the world. As a plant hormone, abscisic acid (ABA) plays an important role in Cd stress response, but its effect on plant Cd uptake and translocation varies among plant species. In some species, such as *Arabidopsis thaliana*, *Oryza sativa, Brassica chinensis*, *Populus euphratica*, *Lactuca sativa*, and *Solanum lycopersicum*, ABA inhibits Cd uptake and translocation, while in other species, such as *Solanum photeinocarpum* and *Boehmeria nivea*, ABA severs the opposite effect. Interestingly, differences in the methods and concentrations of ABA addition also triggered the opposite result of Cd uptake and translocation in *Sedum alfredii*. The regulatory mechanism of ABA involved in Cd uptake and accumulation in plants is still not well-established. Therefore, we summarized the latest studies on the ABA synthesis pathway and comparatively analyzed the physiological and molecular mechanisms related to ABA uptake, translocation, and detoxification of Cd in plants at different ABA concentrations or among different species. We believe that the control of Cd uptake and accumulation in plant tissues can be achieved by the appropriate ABA application methods and concentrations in plants.

## Introduction

An increasing amount of heavy metals have been released into the environment on account of anthropogenic activities, and these heavy metals are posing a serious impact on crop yields, ecosystem functions, and human health, thus causing significant expense to the global economy (Vareda et al., 2019). Among the heavy metals, cadmium (Cd) ranks as one of the top toxic substances, owing to its high mobility, biotoxicity, and prevalence (Zhang et al., 2020). Cd is prone to accumulate in plants through root uptake and further threatens human health through the food chain (Wang et al., 2017). To date, Cd contamination of cropland and crops has received increasing attention worldwide. Therefore, measures are urgently needed to reduce the serious problems caused by Cd pollution.

Over the past few decades, numerous reports on the uptake, translocation and detoxification of Cd in plants have been published, covering crops such as potato (Ye et al., 2020), sweet potato (Huang et al., 2019), maize (Mahmoud et al., 2021), and rice (Yang J. et al., 2020), as well as model plants such as *Arabidopsis thaliana* (Szopiński et al., 2020) and tobacco (Siemianowski et al., 2014). There are various methods to control Cd accumulation in plants, including: (1) Agronomic methods (through plant rotation or co-cropping, etc.) (Rizwan et al., 2016); (2) Genetic engineering (by altering the expression of certain genes) (Wang et al., 2019; Raza et al., 2020); (3) Pollution safe cultivars (by screening low Cd accumulation cultivars) (Huang et al., 2017; Wang et al., 2021); (4) Soil remediation strategy (through the application amendments or physicochemical methods to control the Cd bioavailability) (Hamid et al., 2020; Bilias et al., 2021). For the better implementation of these methods, it is valuable to study the molecular mechanisms related to Cd uptake and accumulation in plants.

Abscisic acid (ABA), a water and ether soluble hormone, was first identified in potatoes and shown to inhibit the growth of buds (Hemberg, 1949). It was later found that ABA can control the abscission of cotton fruits, which is the origin of the name of ABA (Addicott et al., 1968). Recent studies have established that ABA is closely related to abiotic stresses such as drought (Lim et al., 2015), salinity (Hussain et al., 2021), nutrient deficiency (Cutler et al., 2010), and heavy metals (Hu et al., 2020). According to certain studies, ABA was found to promote the uptake and translocation of Cd in plants (Wang et al., 2016; Liu L. et al., 2017; Chen et al., 2021), while other studies showed that ABA inhibited Cd uptake and translocation (Tao et al., 2019; Zhang P. et al., 2019; Leng et al., 2021). To date, the mechanism of ABA in response to the Cd stress of plants is still not well-established. Therefore, it is crucial to reveals some insights into the regulatory mechanism of ABA on Cd uptake and translocation in plants. Although considerable reviews on ABA and heavy metal stress in plants are available, reviews addressing the effects of ABA on plant Cd uptake and accumulation are still rare. Here, we mainly summarize the latest studies on ABA regulatory pathways, the molecular and physiological mechanism of ABA’s effect on plant Cd uptake and translocation, as well as the functions of ABA in plant Cd stress response. It is expected that a reasonable explanation can be put forward for the different effects of ABA on plants Cd uptake and translocation. Also, we are awaiting this review to provide help for the strategies on low-Cd crops cultivation and the phytoextraction technology of soil remediation.

## Synthesis, metabolism, and transport of abscisic acid

**FIGURE 1:**
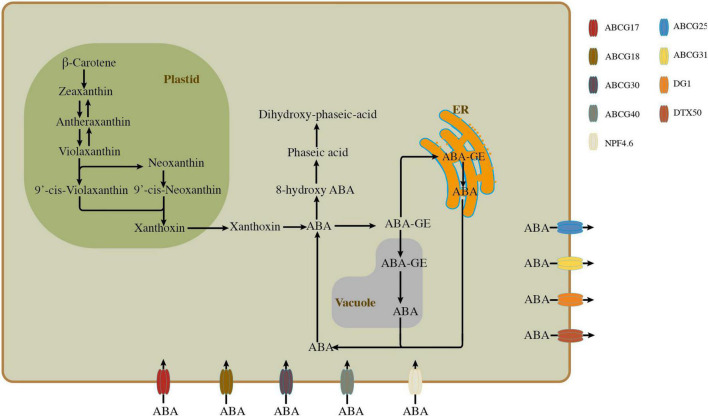
Overall scheme for abscisic acid (ABA) biosynthesis, catabolism, and transport in plant. The pathways of ABA biosynthesis, catabolism, and directions of ABA transport are shown with black arrows. The ABA transporter proteins located on the plasma membrane are represented by different colors.

Abscisic acid is one of the secondary metabolites of plants, which is synthesized from carotene through the interaction of various enzymes along multiple regulatory pathways. Zeaxanthin is an important intermediate for carotenoids to generate ABA and can be synthesized in two pathways (Vishwakarma et al., 2017; Chen et al., 2020; Figure 1). The first pathway is the conversion of β-carotene to zeaxanthin by β-carotene hydroxylase, and the second is the reversible reaction of converting violaxanthin to antheraxanthin and then to zeaxanthin. With the catalysis of zeaxanthin epoxidase (ZEP), zeaxanthin could be converted to all-*trans*-violaxanthin. The downstream products of all-*trans*-violaxanthin are divided into two pathways, one is catalyzed by an indeterminate enzyme to produce 9′-*cis*-violaxanthin, the other is that all-*trans*-violaxanthin is enzymatically converted into *trans*-neoxanthin under the process of ABA-deficient 4 (ABA4) and subsequently transformed to 9′-*cis*-neoxanthin with the help of another uncertain enzyme (Schwartz et al., 1997). Under the oxidative cleavage of 9-*cis*-epoxycarotenoid dioxygenase (NCEDs), both 9-*cis*-neoxanthin and 9-*cis*-violaxanthin are modified to xanthoxin and transported out of the plastid. In cytoplasm, the synthesis of ABA takes place in only two steps, the first step is to reform xanthoxin into ABA-aldehyde at the present of ABA-deficient 2 (ABA2), and the second step is to alter ABA-aldehyde into ABA through the mediation of ABA-aldehyde oxidase 3 (AAO3) (Bittner et al., 2001).

Regarding the metabolic pathway of ABA, it is relatively simple. The surplus ABA can be inactivated and preserved in vacuole and endoplasmic reticulum through the formation of ABA-glucose ester, a process catalyzed by UDP-glucosyltransferase (UGT) encoded by *UGT71C5* (Liu et al., 2015). Also, when ABA deficiency occurs, the preserved ABA-glucose ester in vacuole and endoplasmic reticulum gets activated and released into the cytoplasm *via* β-glucosidases, which is encoded by *Arabidopsis thaliana*β*-glucosidase* (*AtBG1* and *AtBG2*), respectively. In addition to being inactivated and preserved, ABA can also be converted into phaseic acid (PA), then to dihydrophaseic acid (DPA), and finally to DPA-4-O-β-D-glucoside (DPAG) at the presence of cytochrome P450 monooxygenase (P450), PA reductase (PAR), and glycosyltransferase (GT), respectively (Vishwakarma et al., 2017; Chen et al., 2020; Figure 1).

Abscisic acid transportation is often observed in plants to regulate plant growth and development or in response to various stresses. The long-distance transport of ABA between the shoot and root of plants is achieved through xylem and phloem (Kuromori et al., 2014). The xylem is mainly responsible for the transport of ABA from root to shoot, while the phloem accountable for ABA transport in the opposite direction, and the amount of ABA transported by the xylem is considerably higher than that of the phloem (Peuke, 2016; Anfang and Shani, 2021). Short-distance transport of ABA happens mainly between cells, including vascular cells and guard cells, etc. The identification of a number of ABA transporters contributes to the investigation of ABA transport mechanisms. In plants, ABA transporters such as ABCG25 (ATP-binding cassette transporters), ABCG31, DG1 (multidrug and toxin efflux transporter), and DTX50 (multidrug and toxin efflux transporter) are responsible for the efflux of ABA, while ABCG17, ABCG18, ABCG30, ABCG40, and NPF4.6 (nitrate transporter 1/peptide transporter) are involved in the import of ABA (Kang et al., 2010, 2015; Kuromori et al., 2010; Kanno et al., 2012; Zhang et al., 2014; Anfang and Shani, 2021; Figure 1).

Cd stress is similar to other abiotic stresses, such as drought and ultraviolet rays, etc., which can induce the level of endogenous ABA synthesis in plants (Guo et al., 2019). Plants rely on ABA to mimic the impacts of stressful situations and may continuously modify the endogenous ABA contents in response to environmental stresses (Vishwakarma et al., 2017). In addition, the effects of ABA on plant Cd uptake, translocation, and Cd tolerance have been investigated in numerous studies by applying exogenous ABA (Wang et al., 2013; Tao et al., 2019; Hu et al., 2020). Therefore, the molecular and physiological mechanisms of the effects of ABA on plant Cd uptake, translocation and tolerance will be discussed below.

## Abscisic acid affects Cd uptake by regulating signaling pathways and root development

Root uptake is the most important way for the entry of Cd into plants, and ABA manages to influence Cd accumulation in plants by affecting the Cd uptake of root (Yang Y. et al., 2020; Shen et al., 2022). Through the review of numerous studies, it was demonstrated that the effect of ABA on Cd uptake of plant roots is not constant (Table 1). For some plant species and cultivars, ABA significantly promotes Cd uptake, while for other species and cultivars, it exhibits an inhibitory effect on Cd uptake. In addition, the difference in the concentration of ABA treatment might either promote or inhibit the uptake of Cd in some specific plant species and cultivars (Table 1).

**TABLE 1:** Effect of abscisic acid (ABA) on Cd uptake capacity of different species.

Species	ABA sources	Effects on Cd uptake	Mechanisms	References
*Oryza sativa*	Exogenous (up)	Reduced	Reduced transpiration rate	Hsu and Kao, 2003 Uraguchi et al., 2009
	Glutathione-producing bacteria (up)	Reduced	Unknown	Jan et al., 2019
*Arabidopsis thaliana*	Exogenous (up)	Reduced	Inhibiting transcription of IRT1	Fan et al., 2014 Pan et al., 2020
	Exogenous (up)	Reduced	Up-regulates the expression of ABI5	Zhang W. et al., 2019
	Overexpression of MhNCED3	Reduced	down-regulation of IRT and NRAMP	Zhang W. et al., 2019
	ABA-generating bacteria (up)	Reduced	Inhibiting transcription of IRT1	Xu et al., 2018
	ABA-catabolizing bacteria (down)	Enhanced	Mediated HM transporter	Lu et al., 2020a
*Brassica napus*	Exogenous Endogenous	Reduced Enhanced	Effect on the activity of the proton pump	Wang et al., 2018
*Vigna radiata*	ExogenousU (up)	Reduced	Unknown	Leng et al., 2021
*Sedum alfredii*	Exogenous (up)	Enhanced	Up regulation of HMA2 and HMA4	Chen et al., 2022 Lu et al., 2020b
	Plant-growth promoting bacteria (up)	Enhanced	Induced lateral root formation	Wu et al., 2020
	ABA-catabolizing bacterium (down)	Enhanced	Regulated the expression of Cd transporters	Du et al., 2022
	Exogenous (up)	Reduced	Regulated the development of apoplastic barriers in roots of NHE and reduced transpiration	Tao et al., 2017, 2019, 2021
*Brassica chinensis*	ABA-generating bacteria (up)	Reduced	Alleviated the Cd-induced photosynthesis inhibition and oxidative damage	Pan et al., 2019
*Solanum photeinocarpum*	Exogenous (up)	Enhanced	Increased chlorophyll content and biomass	Wang et al., 2016
*Populus euphratica*	Exogenous (up)	Reduced	Restricting Cd2+ Influx	Han et al., 2016
*Lactuca sativa*	Exogenous (up)	Reduced	Increased photosynthesis and antioxidant levels	Tang et al., 2020
	Exogenous (up)	Reduced	Inhibited H_2_O_2_ accumulation and promoted photosynthesis	Dawuda et al., 2020
*Boehmeria nivea*	Exogenous (up)	Enhanced	Unknown	Chen et al., 2021
Zea mays	Cd tolerant bacterium (up)	Reduced	Increased of ABA levels and reduced zmZip expression	Zhou et al., 2019
*Solanum lycopersicum*	Endogenous (up)	Reduced	Unknown	Pompeu et al., 2017
*Bidens pilosa*	Endogenous (up)	Enhanced	Unknown	Liu L. et al., 2017

It was shown that the application of ABA providing a feasible solution for the reduction of Cd uptake in plant roots. When subjected to 1.5 mM of Cd treatment, the endogenous ABA concentration was considerably elevated in Cd-tolerant *Oryza sativa* (rice) cultivar (TNG67), but no significant changes were observed in the Cd-sensitive rice cultivar (TN1). And the increase of endogenous ABA biosynthesis level can reduce the Cd uptake of rice (Hsu and Kao, 2008). After pretreated with 5 mM ABA, Cd accumulation in TN1 was significantly reduced by 44%, whereas no significant change of Cd accumulation was observed in TNG67 (Hsu and Kao, 2003). The application with 10 μM of ABA not only markedly reduced (*p* < 0.05) the Cd contents in the roots and stems of *Vigna radiata* (mung bean) seedlings by 23.5 and 25.3% respectively, but also significantly elevated (*p* < 0.05) the chlorophyll and carotenoid content (Leng et al., 2021). In species such as *Brassica chinensis*, *Populus euphratica*, *Lactuca sativa*, and *Solanum lycopersicum*, ABA content was also negatively correlated with the Cd accumulation levels (Han et al., 2016; Pompeu et al., 2017; Pan et al., 2019; Tang et al., 2020). Only the mechanisms of *Brassica chinensis* and *Populus euphratica* were explicated as that ABA elevated antioxidant enzyme activity and reduced the intracellular H_2_O_2_ content by enhancing the antioxidant capacity of plants, which was responsible for regulating the entry of Cd through the calcium ion channel, thus leading to a reduction in the level of Cd uptake (Han et al., 2016; Pan et al., 2019). However, in *Solanum photeinocarpum* and *Boehmeria nivea*, ABA enhanced the Cd uptake mainly by increasing the chlorophyll level and biomass of the plants (Wang et al., 2016; Chen et al., 2021). Apart from species, different ABA concentrations showed different effects on the Cd uptake capacity of the same plant. In *Boehmeria nivea* and *Solanum photeinocarpum*, the strongest Cd uptake was observed at the application of 5 and 20 μM of ABA, respectively, however, the relevant mechanisms involved are not clearly explored (Wang et al., 2016; Chen et al., 2021). Although application of ABA also promoted Cd uptake of *Bidens Pilosa*, it diminished the biomass and chlorophyll content (Liu L. et al., 2017), which was the exact contrast to its effect on stimulating the growth of *Solanum photeinocarpum* and *Boehmeria nivea* and other spices.

Therefore, further studies are needed to fully reveal the mechanism by which ABA affects Cd uptake among species, and whether ABA has any effect on root Cd uptake by impacting root development also needs to be investigated.

### Molecular mechanism of abscisic acid affecting Cd uptake among species

**FIGURE 2:**
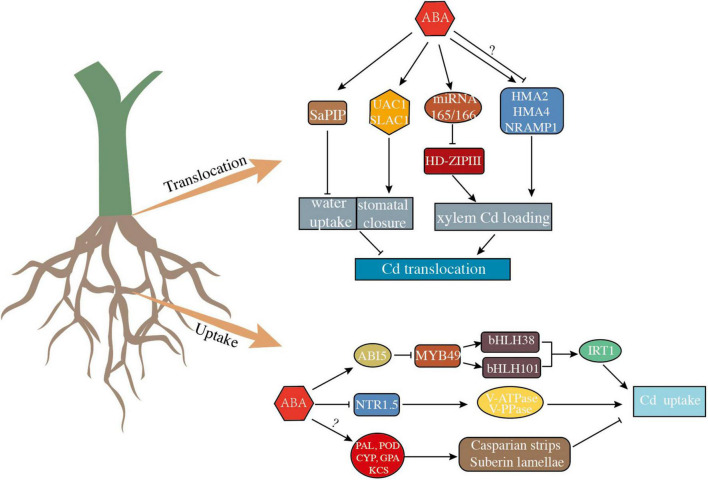
A proposed model of the effect of abscisic acid (ABA) on Cd uptake and translocation in plants. Arrows indicate positive regulation of ABA and its corresponding targets, bars indicate negative regulation of ABA and its corresponding targets, question marks represent regulatory pathways that remain unclear.

As a model plant, *Arabidopsis thaliana* has been well-studied for the ABA effect on Cd uptake and accumulation. Firstly, application of exogenous ABA elevated the ABA levels in *Arabidopsis thaliana* and simultaneously suppressed the expression of iron-regulated transporter 1 (*IRT1*), a key transporter responsible for Cd uptake in roots, thereby leading to the reduction of Cd uptake and accumulation in *Arabidopsis thaliana* (Vert et al., 2002). Under Cd stress, ABI5 could bind to the transcription factor MYB49 and block the function of the bHLH38 and bHLH101 promoters that required for *IRT1* expression, thereby reducing IRT1 concentration (Wu et al., 2012; Wang et al., 2013). Zhang et al. (2019) revealed that the application of ABA stimulated the expression of *ABI5* and thus reduce the Cd uptake in *Arabidopsis thaliana* by regulating the expression level of IRT1. ABA-importing transporters (AIT1) contributed to the entry of ABA into the plant roots and then inhibited the expression of *IRT1*, thus limiting the uptake of Cd of *Arabidopsis thaliana* (Pan et al., 2020). Furthermore, inoculation with ABA-generating bacteria strains and ABA-catabolizing bacteria strains resulted in corresponding increases and decreases of ABA levels in *Arabidopsis thaliana*, respectively, which in turn inhibited or promoted Cd uptake and accumulation by affecting the expression levels of *IRT1* in *Arabidopsis thaliana* (Xu et al., 2018; Lu et al., 2020a). In addition, some transgenes and mutant experiments on *Arabidopsis thaliana* have investigated the functions of ABA synthesis-related genes or ABA signaling-related genes in Cd uptake and accumulation. Multiple studies using *Arabidopsis thaliana* transgenic plants and mutants have demonstrated that ABA synthesis-related genes (*ABA-1*, *ABA-3*, *ABA-4*, and *nced3*) and ABA signaling-related genes (*ABI2*, *ABI3*, *ABI5*, *SnRK2s*, and *PP2Cs*) are involved in Cd uptake and accumulation (Umezawa et al., 2009; McLoughlin et al., 2012; Zhang et al., 2019). In general, the accumulation of Cd in *Arabidopsis thaliana* was negatively correlated with the concentration of ABA (Figure 2).

In *Sedum alfredii*, ABA showed opposite effects on plant Cd uptake in different experiments. On one hand, ABA inhibited the uptake of Cd, unlike non-hyperaccumulating ecotype (NHE) in which the endogenous ABA content was significantly elevated, the endogenous ABA in hyperaccumulating ecotype (HE) remained at a relatively minimum level in response to Cd stress. The elevated endogenous ABA level should be attributed to the significant up-regulation of *ABA2* and *NECD* expression levels of NHE under Cd treatment (Tao et al., 2019, 2021). Studies firmly indicate that the root apical zone is the most dynamic part for Cd uptake (Lux et al., 2011; Redjala et al., 2011). Casparian strips (CSs) and suberin lamellae are important barriers preventing Cd from entering the plant root *via* the apoplastic pathway (Vaculík et al., 2009; Ricachenevsky et al., 2018). The distance from the CSs to the root tip (L_TIP_-CSs) and the proportion of the suberin lamellae deposited in the root were significantly related to the ability of Cd uptake by plants. Studies have shown that ABA exerts a catalytic effect on suberin biosynthesis and deposition (Barberon et al., 2016; Tao et al., 2017). By confocal laser scanning microscope, Tao et al. (2019) found that the L_TIP_-CSs and non-suberized portions in the root were statistically inversely correlated with the root ABA content, indicating that ABA reduced Cd uptake by regulating the development of apoplastic barriers in roots of NHE. ABA may enhance the deposition of CSs and suberin by elevating the expression of *PAL*, *POD*, *SaCYP86A1*, *SaGPAT5*, and *SaKCS20* that related to CSs and suberin synthesis in *Sedum alfredii*. Du et al. (2022) demonstrated that ABA-catabolizing bacterium *R. qingshengii* was able to enhance the expression of Cd uptake related transporters by reducing the ABA content in *Sedum alfredii*, thus enhancing the Cd uptake capacity. On the other hand, it is interesting to note that the study of Chen et al. (2022) and Lu et al. (2020b) on Cd uptake of *Sedum alfredii*. Conflicted with the previous studies. It was found that Cd uptake of *Sedum alfredii* was significantly enhanced by foliar spraying of ABA, which was attributed to the fact that ABA enhanced the expression of *HsfA4c* and *NAS* thus promoted the tolerance and uptake of Cd by plants. Such a contradictory situation of *Sedum alfredii* should ascribe to the difference in ABA application methods and concentrations (Figure 2).

Application of exogenous ABA inhibited the Cd uptake in *Brassica napus*, which was due to the inhibition of *NTR1.5* (a long-distance transporters of NO_3_^–^) expression by ABA, causing a decrease in proton pump activity of V-ATPase and V-PPase, which resulted in lower Cd uptake in the roots (Wang et al., 2018). It is intriguing that endogenous ABA in *Brassica napus* is just the contrary of exogenous ABA, which affects the function of proton pump to enhance root Cd accumulation, but the specific mechanism still needs to be further investigated. Cd tolerant bacterium NC16 could also elevate the ABA content and subsequently diminish the Cd uptake of *Zea mays* by inhibiting the expression of a zinc/iron transporter (Zhou et al., 2019; Figure 2).

### The effects of abscisic acid on Cd uptake through regulating root development

The root system is the main passage for Cd to enter plants, therefore, the relationships between Cd uptake and root morphology among different plant species have been widely investigated (Ge et al., 2016; Deng et al., 2020; Guo et al., 2020). Variations in root morphology and structure may affect Cd uptake and xylem loading, leading to differences in the Cd accumulation and distribution among plant tissues (Vaculík et al., 2012). In the hyperaccumulating ecotype of *Sedum alfredii*, the root length, area, and volume were improved under Cd treatment, which exhibited a significant and positive correlation with its Cd uptake and accumulation (Li et al., 2009). The comparative study of high- and low-Cd cultivar of pakchoi, tomato, hot pepper, peanut, and wheat revealed that high-Cd cultivar possessed longer root length, more root tips, larger root surface area, and greater root volume, which should contribute to its higher Cd uptake and translocation (Kubo et al., 2011; Zhang et al., 2013; Huang et al., 2015; Xia et al., 2016; Xin et al., 2017). Through the study of 18 herbaceous plants, Yu et al. (2017) observed that longer and slimmer roots facilitated the translocation of Cd from to shoot, while coarser roots (diameter of 0.6–0.8 mm) contributed to the immobilization of Cd in roots and thus inhibited its translocation to shoot.

According to the review of Harris (2015), in which ABA was ascribed as “Hidden Architect of Root System Structure,” indicating the crucial role of ABA in root development. ABA regulates root development mainly by working on stem cells and controlling the activity of quiescent centers in the root meristem, and it has been widely accepted that low concentrations of ABA promote root development, while high concentrations inhibit root development (Sun et al., 2018; Brookbank et al., 2021). The root growth promotion at low concentration of ABA was mainly achieved through the regulation of auxin pathway and auxin efflux carrier (PIN2/EIR1), while the root inhibition by high concentration of ABA was mediated by the combination of auxin efflux protein (AUX1) and ethylene pathway (Li et al., 2017; Brookbank et al., 2021). In addition, ABA is also observed to inhibit root development by evoking ROS and elevating cytoplasmic Ca^2+^ content in root (Jiao et al., 2013; Tsukagoshi, 2016). However, the sensitivity of different plants to ABA varies according to their growth environment and genetic diversity, therefore the same concentration of ABA may promote root development in some plants but inhibit root development in others. Concentration of ABA amounting to 1 μM was sufficient to inhibit lateral root (LR) development in *Arabidopsis thaliana*, whereas for *M. truncatula*, ABA concentrations in the range of 0.1–10 μM were found to promote LR development, and LR formation was inhibited only at ABA concentrations above 50 μM (Ariel et al., 2010; Gonzalez et al., 2015). According to the study of Chen et al. (2006), ABA is able to promote lateral root production in rice like it does in most legumes including *M. truncatula*, and the calcium, calmodulin and *de novo* protein synthesis are required during the stimulation process.

Through statistical analysis of studies related to plant root development and Cd uptake capacity, we discovered that the level of Cd accumulation in plants under the effect of ABA was not necessarily positively correlated with their root development. In some plant species, ABA is capable of enhancing the level of root development, while reducing the ability of plants to accumulate Cd. The application of 0.5 μM ABA increased root biomass by 16%, but resulted in a 27% decrease in Cd uptake and accumulation in *Arabidopsis thaliana* (Fan et al., 2014; Pan et al., 2020). The use of 10 μM ABA also significantly increased root biomass and decreased Cd content in mung bean (Leng et al., 2021). Besides, abscisic acid-deficient sit tomato mutant showed stunted root development and significantly increased Cd content (Pompeu et al., 2017). When co-cultured with ABA-generating bacteria, biomass was significantly increased and Cd content was also decreased in pakchoi (Pan et al., 2019). In *Sedum alfredii*, the higher ABA concentration (0.2 mg/L) inhibited the root biomass, but promoted the uptake and accumulation of Cd (Lu et al., 2020b). However, in other plants, such as ramie (*Boehmeria nivea* L.), purple flowering stalk and *Solanum poteinocarpum*, lower level of ABA can simultaneously promote root development and Cd uptake and accumulation (Wang et al., 2016; Shen et al., 2017; Chen et al., 2021). In addition, the significantly root biomass improvement and higher Cd uptake capacity were observed in *Sedum alfredii* with the application of ABA-catabolizing bacterium *Rhodococcus qingshengii* (Wu et al., 2020; Du et al., 2022).

Therefore, ABA affects the development of plant roots through auxin and ethylene regulatory pathways, and a large number of studies have shown that the development of plant roots helps plants to improve Cd uptake to a certain extent. However, the effect of ABA on plant Cd uptake capacity is not only achieved by the level of root development, but also depends on various factors such as the effect of ABA on the expression level of Cd-related transporters.

## Abscisic acid influences Cd translocation by regulating transpiration, xylem loading, and Cd transporters

In general, the effect of ABA on plant Cd translocation is basically similar to its effect on plant Cd uptake capacity. To date, ABA is known to regulate Cd translocation in plants in three ways: (1) affecting plant transpiration; (2) xylem loading (apoplast pathway); (3) regulating the expression of Cd transporters (Mendoza-Cózatl et al., 2011; Zhao and Wang, 2020; Khanna et al., 2022).

Transpiration pull is the main driving force for Cd transport from roots to shoots in plants, and studies have shown that the difference in Cd translocation among different plants is mediated by the changes of transpiration rates (Uraguchi et al., 2009; Khanna et al., 2022). Plants have been found to regulate transpiration by controlling root water uptake (Cai et al., 2022), regulating xylem conduits (Schenk et al., 2021) and moderating the density and closure of stomata (Agurla et al., 2018; Tao et al., 2021). ABA was revealed to regulate plant stomatal closure through kinase-mediated phosphorylation of guard cell membrane-localized transporters, such as R-type anion channel QUAC1 and S-type anion channel SLAC1, which should hinder the translocation of metal ions from root to shoot (Pornsiriwong et al., 2017; Hsu et al., 2021). According to the study of Kuromori et al. (2016), overexpression of ABA transporter (*AtABCG25*) in *Arabidopsis thaliana* resulted in stomatal closure and reduced transpiration. In *Sedum alfredii*, ABA regulates the transpiration rate by decreasing the expression of root aquaporin (*SaPIP*), stomatal density and size, thereby leading to the reduction of Cd translocation from root to shoot (Tao et al., 2021). Likewise, transpiration in rice was reduced by 64–72% under 100 mM ABA treatment, while the shoot Cd content was correspondingly reduced by about 10-fold (Hsu and Kao, 2003). When pretreated with 5 μM ABA, Cd concentrations in shoots of *Phytolacca americana* were significantly reduced, which was attributed to the inhibition of transpiration (Liu et al., 2010). In lettuce, studies have also shown that exogenous ABA can reduce Cd transport by inhibiting transpiration (Aroca et al., 2008; Dawuda et al., 2020). Therefore, it is speculated that ABA mainly regulates Cd translocation in plants by inhibiting transpiration, however, most of the studies are only at the physiological level, and the related molecular mechanisms remain to be investigated.

Root xylem loading of Cd is one of the most important steps for its translocation to shoot, and the Cd concentration in xylem sap is significantly and positively correlated with Cd concentration of shoot (Uraguchi et al., 2009; Mendoza-Cózatl et al., 2011; Song et al., 2017). The miRNA165 and miRNA166 are known signaling molecules that determine the development of xylem cells in plant stele (Carlsbecker et al., 2010; Miyashima et al., 2011). In endodermis, ABA elevated the expression level of miRNA165, which in turn negatively affected HD-ZIPIII TF (transcription factors in the stele) levels and led to higher xylem hydrophobicity (Ryu et al., 2016; Ramachandran et al., 2018). According to the study of Bloch et al. (2019) in *Arabidopsis thaliana* and tomato, ABA was revealed to affect HD-ZIPIII levels by upregulating miRNA165a/166b expression, enhancing xylem lignin deposition, and limiting lateral root formation and elongation, which apparently reduced Cd loading into xylem of the plant root. Moreover, the master regulators for xylem differentiation, VASCULAR-RELATED NAC DOMAIN (VND) 1-3 and 7, are regulated by ABA in response to environmental stress (Ramachandran et al., 2021). Strikingly, the reduction of miRNA165/166 levels in *Arabidopsis thaliana* plants by using artificial miRNA-target (STTM165/166) resulted in elevated expression of ABA and its related genes (Yan et al., 2016), whereas application of STTM166 did not affect ABA content in rice (Zhang et al., 2018), suggesting the signaling pathways and functions of miRNA166 are inconsistent between rice and *Arabidopsis thaliana*. Furthermore, ABA was observed to have no significant effect on plant xylem vessel length and diameter, but it greatly reduced the vessel count of xylem, limiting Cd loading and transpiration, thereby inhibiting Cd transport to shoot (Campbell et al., 2018; Tao et al., 2021). Studies on ABA regulation of xylem differentiation and Cd loading are relatively rare, therefore the future studies concerning the effect of ABA on Cd transport in plants through regulation of miRNA166 and VND will be interesting.

Membrane transporters serve a critical function in the loading of Cd into root vascular tissue and subsequent translocation in the plants (Zhao and Wang, 2020). ABA was shown to induce the expression of a number of Cd transporters, thereby influencing Cd translocation in plants (He et al., 2020). The heavy metal ATPases HMA2 and HMA4, localized on the plasma membrane, contribute to the xylem Cd loading, thus facilitating the translocation of Cd from root to shoot (Papoyan and Kochian, 2004; Takahashi et al., 2012). NRAMP1 (resistance-associated macrophage protein 1) contributes to the translocation of Cd from root to shoot as well (Yang Y. et al., 2020). In contrast, HMA3, localized on the tonoplast, is able to promote the sequestration of Cd in vacuole and inhibit the translocation of Cd in plants (Liu H. et al., 2017; Begum et al., 2019). However, there are few studies on Cd transporters involved with ABA treatment showed inconsistent results. Chen et al. (2022) and Lu et al. (2020b) demonstrated that endogenous ABA promoted the loading of Cd into the xylem by inducing the expression of *HMA2* and *HMA4*, thus elevating the Cd content in the shoot of *S. alfredii* and leading to increased Cd translocation factor (TF) values. The transcription factor NAC895 was able to elevate the loading of Cd into the xylem by promoting the expression of *HMA2* in the presence of ABA (Liu et al., 2022). Under the gradient treatment of ABA from 0 to 40 μmol/L, the TF values of Cd were maximally elevated by 16.8 and 11.4% in *Solanum photeinocarpum* and *Bidens Pilosa*, respectively, however, the specific mechanism by which ABA enhanced the Cd translocation was not interpreted (Wang et al., 2016; Liu L. et al., 2017). Conversely, ABA-induced reduction of Cd translocation in Arabidopsis can be achieved through overexpression of *MhNCED3* and inhibition of *HMA2* and *NRAMP1* expression levels (Pan et al., 2019). When cultured with ABA-degrading bacteria, the expression of *HMA2*, *HMA3*, and *HMA4* were up-regulated and resulted in elevated Cd content in the shoot of *Arabidopsis thaliana* (Lu et al., 2020a).

In conclusion, the role of ABA on Cd translocation by reducing transpiration and decreasing xylem length, number and hydrophilicity is relatively clear and consistent, but the function of ABA in regulating the expression of Cd transporters varies widely. We propose that the translocation of Cd should be achieved through the combination of multiple aspects, rather than explained in only one aspect. Therefore, some indepth research work is still needed to investigate how ABA regulates the translocation of Cd from roots to shoots (Figure 2).

## Abscisic acid enhances Cd tolerance by regulating antioxidant systems

Reactive oxygen species (ROS), including superoxide anion (O_2_^–^), hydrogen peroxide (H_2_O_2_), singlet oxygen (^1^O_2_), and hydroxyl radical (OH•), is a kind of oxygen-containing molecules, which are mainly produced by transferring electrons to molecular oxygen (O_2_) during photosynthesis and aerobic respiration in plants (Qi et al., 2017; Waszczak et al., 2018). ROS acts as signaling molecules, which participate in plant growth, development, and adaptation to biotic and abiotic stresses (Baxter et al., 2014; Choudhury et al., 2017). However, heavy metals (such as Cd) will lead to the surge of ROS, which can cause oxidative damage to cellular biological macromolecules (including lipid, proteins, DNA, and RNA), even resulting in cell death (Mittler, 2002; Shahid et al., 2014; Hu et al., 2016). Therefore, maintaining ROS homeostasis has great importance for plants to adapt to stresses (Miller et al., 2010).

Abscisic acid (ABA) plays key roles in plant tolerance to multiple stresses, including salinity, drought, and heavy metals (Bari and Jones, 2009; Hauser et al., 2017; Mega et al., 2019; Hu et al., 2020; Tao et al., 2021). Many studies showed that Cd stress promoted the production of endogenous ABA, and exogenous ABA increased Cd tolerance through scavenging Cd-induced ROS by regulating antioxidant enzymes and/or non-enzymatic constituents in plants. In tomato (*Solanum lycopersicum*), the ABA-deficient mutant was less tolerant to Cd stress and displayed higher antioxidant activity than its wild type, suggesting that the responses to Cd stress in tomato was enhanced by ABA deficiency (Pompeu et al., 2017). Exogenous ABA application reduced malondialdehyde (MDA), H_2_O_2_, and O_2_^–^ contents in purple flowering stalk (*Brassica campestris* L. ssp. chinensis) by activating the antioxidant enzymes, including superoxide dismutase (SOD), peroxidase (POD), ascorbate peroxidase (APX), and glutathione reductase (GR), thus relieving Cd toxicity (Shen et al., 2017). In a Cd-sensitive cultivar of lettuce (*Lactuca sativa* L.), foliar application of ABA alleviated Cd-induced oxidative damage by increasing SOD, catalase (CAT), POD activities, and chlorophyll and carotenoids contents (Dawuda et al., 2020). ABA reduced Cd-elicited H_2_O_2_ by increasing CAT, POD, and APX activities, contributing to the improved Cd tolerance of Cd-hypersensitive *Populus euphratica* (Han et al., 2016). Foliar application of ABA increased Cd tolerance of mung bean seedlings [*Vigna radiata* (L.) R. Wilczek] and largely recovered Cd-induced changes in antioxidant systems, including APX, CAT, and POD activities, MDA, proline, and ascorbic acid (AsA) contents (Leng et al., 2021). Under 100 μM Cd treatment, 0.2 mg/L ABA promoted the growth of *Sedum alfredii* Hance (a Cd hyperaccumulator) and reduced its MDA content (Lu et al., 2020b).

Several studies have shown that ABA affects antioxidant enzymes by regulating transcription factors (TFs), such as WRKYs (Hong et al., 2016). WRKYs participate in the signal transduction processes responding to stresses by binding to the WRKY binding sites (W-boxes, C/TTGACC/T) in the promoters of the defense-related genes and could be regulated by ABA (Eulgem and Somssich, 2007; Yang et al., 2016). Several studies have proved that WRKYs are key links in ABA signaling networks and play crucial roles in plant responses to different stresses by maintaining ROS homeostasis (Chen et al., 2010; Rushton et al., 2012; Sun et al., 2022). For instance, the overexpression of *ZmWRKY4* in maize (*Zea mays*) increased the levels and activities of SOD and APX, while the RNAi of *ZmWRKY4* inhibited the increase of SOD and APX expression levels and activities induced by ABA, suggesting that *ZmWRKY4* was necessary for ABA regulating antioxidant enzyme systems under stress (Hong et al., 2016). In *Tamarix hispida*, *ThWRKY4* was highly induced by ABA, drought and salt stresses, and overexpression of *ThWRKY4* decreased ROS (O_2_^–^ and H_2_O_2_) levels in *Arabidopsis thaliana* by increasing SOD and POD activities (Zheng et al., 2013). Over expression of *Tamarix hispida ThWRKY7* significantly enhanced Cd tolerance in *Arabidopsis thaliana* by increasing SOD and POD activities (Yang et al., 2016).

## Conclusion and perspective

In this review, we analyzed and summarized the anabolism of ABA, effect of ABA on the Cd uptake and translocation, correlation between ABA on plant root development and Cd accumulation, and the effect of ABA on ROS mitigation in plants. We believe that the Cd uptake and translocation affected by ABA is closely related to plant species, the application methods and concentrations of ABA. In most cases, ABA serves the inhibitory function of Cd uptake mainly through: (1) regulating the ABI5-MYB49-IRT1 pathway; (2) facilitating the deposition of CSs and suberin lamellae; (3) suppressing the proton pump activity of V-ATPase and V-PPase by inhibiting the expression of NTR1.5. The effect of ABA on Cd translocation is primarily through: (1) inhibiting transpiration by modulating the phosphorylation of QUAC1 and SLAC1, the expression of *SaPIP* and stomatal density and size; (2) elevating xylem hydrophobicity by affecting expression levels of miRNA165/166, HD-ZIPIII TF, and VND; (3) regulating the expression Cd transporters such as HMA2, HMA3, HMA4, and NRAMP1. ABA is capable of affecting root development through auxin and ethylene pathway, but its correlation with plant Cd uptake and accumulation capacity is not significant. Since the effects of ABA on Cd uptake and translocation vary distinctly among species and the concentrations of ABA, future work should focus on: (1) mechanistic differences between endogenous and exogenous ABA on Cd uptake and accumulation in plants; (2) mechanisms underlying the effects of exogenous ABA (including concentration and application method) on the differences in Cd uptake and accumulation among species; (3) application of ABA for low cadmium accumulation in crops to ensure food safety and for high cadmium uptake in phytoremediation.

## Author contributions

CS and Y-YH conceptualized the review, wrote the manuscript, and prepared the figures. Y-MY, Y-FS, MZ, and X-JC helped to collect data and write the manuscript. All authors contributed to the article and approved the submitted version.
